# The complete chloroplast genome of *Senna alata* (L.) Roxb., an important medicinal plant from the Philippines

**DOI:** 10.1080/23802359.2023.2172973

**Published:** 2023-02-10

**Authors:** Kristine J. O. Quiñones, Renerio P. Gentallan, Michael C. B. Bartolome, Roselle E. Madayag, Juan R. A. Vera Cruz, Angeleigh R. T. Cirunay, Leah E. Endonela, Emmanuel B. S. Timog, Teresita H. Borromeo, Nestor C. Altoveros, Bartimeus B. S. Alvaran, Jessabel B. Magtoltol, Reneliza D. Cejalvo

**Affiliations:** a Institute of Crop Science, College of Agriculture and Food Science, University of the Philippines Los Baños, Los Baños, 4031 Laguna, Philippines; b Institute of Food Science, College of Agriculture and Food Science, University of the Philippines Los Baños, Los Baños, 4031 Laguna, Philippines; c Agricultural Systems Institute, College of Agriculture and Food Science, University of the Philippines Los Baños, Los Baños, 4031 Laguna, Philippines; d Department of Forest Biological Sciences, College of Forestry and Natural Resources, University of the Philippines Los Baños, Los Baños, 4031 Laguna, Philippines

**Keywords:** *Cassia alata*, Fabaceae, Cassinae, chloroplast genome, medicinal plant

## Abstract

*Senna alata*, a flowering shrub, is widely cultivated in the Philippines for its anti-fungal properties. Despite this, its chloroplast genome is not yet established. We assembled and annotated the complete chloroplast genome of accession from the germplasm collection of the Institute of Crop Science, University of the Philippines, Los Baños, using Illumina sequencing data. The complete cp genome was 159,176-bp long characterized by a large single copy of 88,769 bp, short single-copy of 18,301 bp and a pair of inverted repeat regions of 26,053 bp each. The overall GC content of the chloroplast genome was 36.4%. The plastome comprised 37 tRNA genes, 8 rRNA genes and 78 mRNA genes. Phylogenetic analysis showed that *S. alata* is closely related to *S. siamea*.

## Introduction

*Senna alata* (Linnaeus) Roxburgh 1832, also known as candle bush, is a flowering ornamental shrub under subfamily Caesalpinioideae of family Fabaceae (Leguminosae). The species is native to Colombia, Venezuela, the Guyanas and Brazil (Irwin and Barneby 1982) and also commonly found in Asia and Africa (Kumar et al. 2008). It was introduced in the Philippines during the Spanish colonial period, ca. 16th–eighteenth century, *via* the Galleon trade (Amano et al. 2021). *S. alata* is locally called “akapulko” in the country, and it is widely dispersed and cultivated for medicinal purposes (Palanichamy and Nagarajan 1990; Philippine Pharmacopeia 1 [PPI], 2004). It has been used to treat diseases due to its antifungal (Oladeji et al. 2016), laxative (Adelowo and Oladeji 2017), anti-helmintic (Kundu et al. 2012) and anti-inflammatory (Sagnia et al. 2014) properties. Akapulko is included in the Department of Health (DOH)-Philippines list of ten recommended medicinal plants that had been validated for their safety and efficacy (Zarsuelo et al. 2018). Despite these promising health-promoting benefits, no investigation has characterized the complete chloroplast genome sequence; hence, in this study, we assembled and annotated the complete chloroplast of *S. alata,* which will be useful in elucidating evolutionary and phylogenetic relationships under the subfamily Caesalpinioideae of the Fabaceae family.

## Materials and methods

We collected fresh leaves of *S. alata* from the field genebank of the Crop Breeding and Genetic Resources Division, Institute of Crop Science, University of the Philippines Los Baños (UPLB), Laguna, Philippines with a type locality at Nueva Ecija, Philippines (15° 22′ 04.0” N 121° 03′ 26.0” E). We characterized the morphology of the accession (ICROPS 2019 149) based on the identified distinguished morphological markers in the *Senna* group (Roxburgh and Wallich 1820) for future taxonomic revisions. This is also to ensure that the genotype is associated with a particular reference specimen conserved in the genebank and preserved in the herbarium. The whole plant, leaves, inflorescence and pods of the accession in the field genebank were also photographed using the Nikon COOLPIX S9500 Digital Camera. The quantitative measurements reported in this paper are computed average values. The voucher specimen (ICROPS 2019 149) of this accession was deposited in the Philippine Herbarium of Cultivated Plants, UPLB (https://cafs.uplb.edu.ph/icrops/, Renerio P. Gentallan Jr., rpgentallan@up.edu.ph). The genomic DNA from fresh samples was extracted using the slightly-modified CTAB method (Doyle and Doyle 1987) and sent to NovogeneAIT Genomics Singapore PTE LTD, Singapore, for sequencing using the HiSeq-PE150 platform (Illumina Inc., San Diego, CA, USA). The 150-bp pair-end raw reads were produced which were successively filtered to generate 18,652,000 of cleaned reads. We used the GetOrganelle v1.7.5+ software (Jin et al. 2020) to assemble the chloroplast genome which generated a circular genome. Subsequently, this genome was annotated and mapped using CPGAVAS2 (Shi et al. 2019) and was visualized using Chloroplast Genome Viewer (CPGView) (Liu et al. 2023). The assembled chloroplast genome sequence was submitted to the GenBank (accession no. ON653612/NC_065665.1) of the National Center for Biotechnology Information (NCBI).

## Results and discussion

### Morphological characterization of the plant material

**Figure 1.**
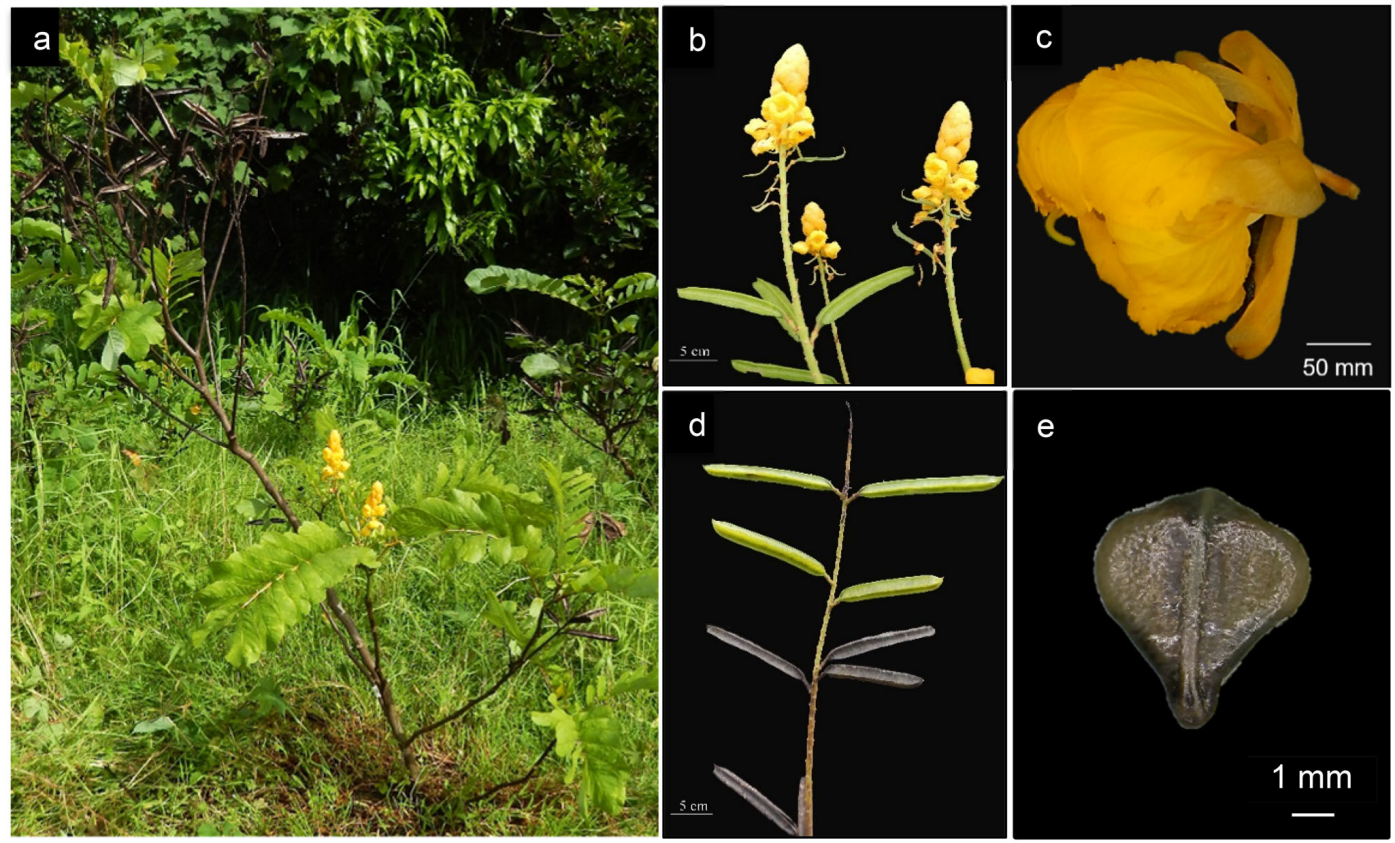
Morphological characteristics of *S. alata* (ICROPS 2019 149) used for chloroplast genome sequencing; whole plant (a), inflorescence (b), floret (c) pod (d) and seed (e). (Photo source: ©CBGR Laboratory, ICropS, CAFS, UPLB).

*S. alata*is 2.29 m tall with a 1.55 m spread. Leaflet (Figure 1a) opposite, 8 pairs, blade obovate, retuse apex, dark green with a yellow midrib and orange margin color, 9.82 × 4.6 cm. Petal (Figure 1b) ovate, 15.02 cm long while bract, pedicel and rachis (Figures 1b and 1c) are 12.48, 6.34 and 37.3 cm long, respectively. Pod (Figure 1d) luster matte with a strong degree of ridge depth, 11.82 × 1.76 cm. Seed (Figure 1e) surface wrinkled, glossy, black, 11.83 × 4.41 × 2.09 mm. The characteristics of the accession (ICROPS 2019-149) used for the assembly fall within the range of the description established for *S. alata* (Roxburgh and Wallich 1820). Hou et al. (1996) reported that *S. alata* is differentiated from other species through its characteristic pedicel that is shorter than the sepal and pods (Figure 1c), which are winged.

### Complete chloroplast genome and phylogenetic anlaysis of S. alata

**Figure 2.**
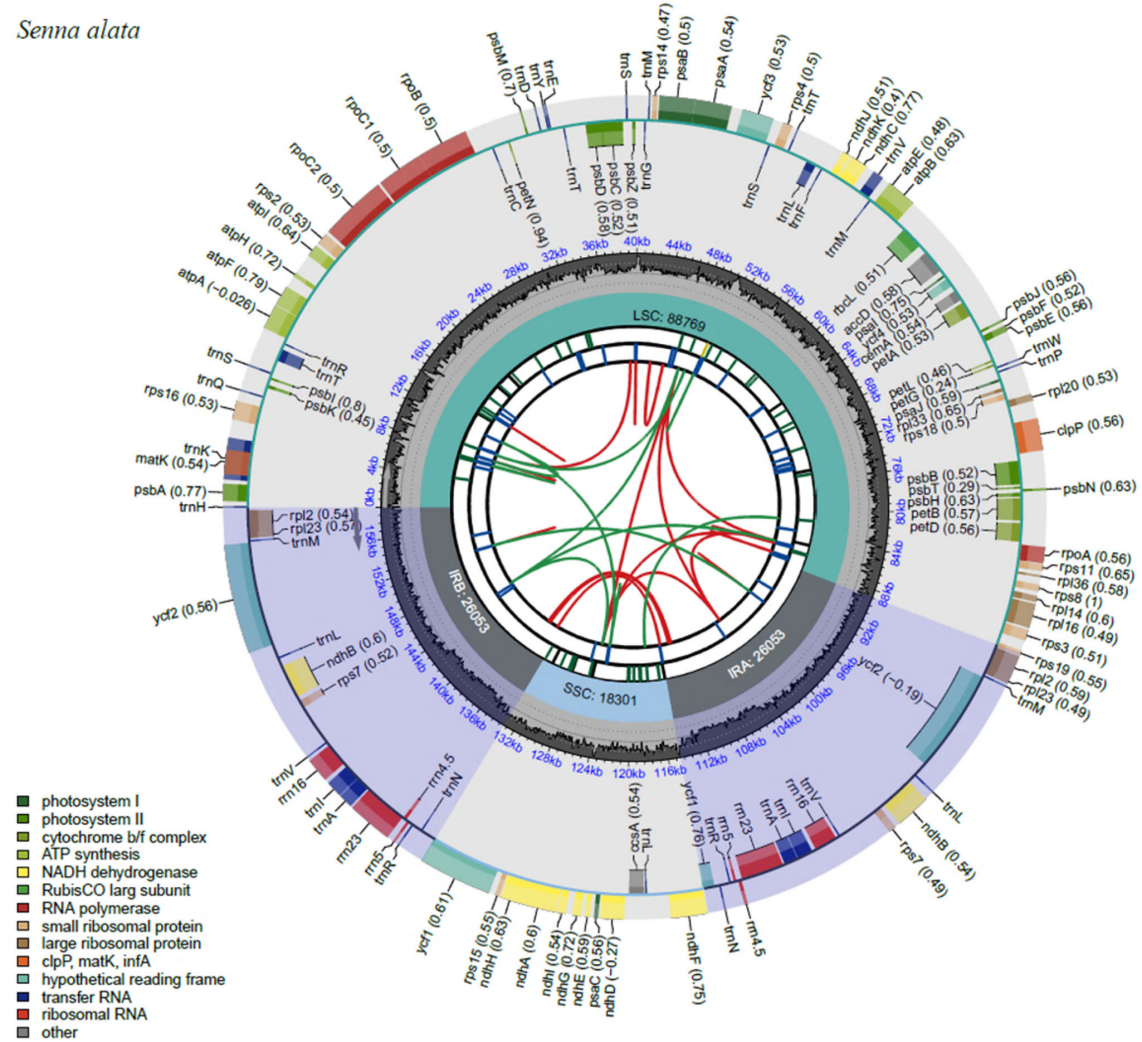
Chloroplast genome map of *S. alata*. The map contains seven circles, where the first circle (from the center going outward) shows the distributed repeats connected with red (the forward direction) and green (the reverse direction) arcs. The second circle shows the tandem repeats marked with short bars. The next circle shows the microsatellite sequences as short bars. The fourth circle shows the size of the LSC and SSC. The fifth circle shows the IRA and IRB while the sixth circle shows the GC contents along the plastome. The seventh circle shows the genes having different colors based on their functional groups.

The chloroplast genome of *S. alata* had a sequence length of 159,176 bp which exhibited a typical quadripartite circular structure (Figure 2, Figure S1). The sequence included a large single copy (LSC), a short single-copy (SSC) and two inverted repeat regions (IRa and IRb) corresponding to 88,769, 18,301 and 26,053 bp, respectively. The overall GC content of the chloroplast genome was 36.4% with base compositions of 31.4% A, 32.2% T, 17.9% G and 18.5% C. *S. alata* had a shorter sequence length compared to *S. bicapsularis* with 161,056 bp (Hou 2020), *S. spectabilis* with 162,754 bp (Shi et al. 2020), *S. tora* with 161,050 bp (Xu et al. 2020), *S. occidentalis* with 159,993 bp (Kang et al. 2020) and *S. obtusifolia* with 162,426 bp (Yu et al. 2020). The chloroplast genome of S. *alata* comprised of 123 genes coding for 37 tRNAs, 8 rRNAs and 78 mRNAs. Among these are 42 genes for photosynthesis, 27 genes for self-replication, three conserved ORF (*ycf1, ycf1, ycf4*), and 6 other genes (*accD, ccsA, cemA, clpP, infA* and *matK*). In addition, 13 cis-splicing genes were observed, out of which 9 were unique (*rps16, atpF, rpoC1, ycf3, clpP, petB, petD, rpl16, ndhA*) and 2 were duplicated (*rpl2* and *ndhB*) (Figure S1).

**Figure 3.**
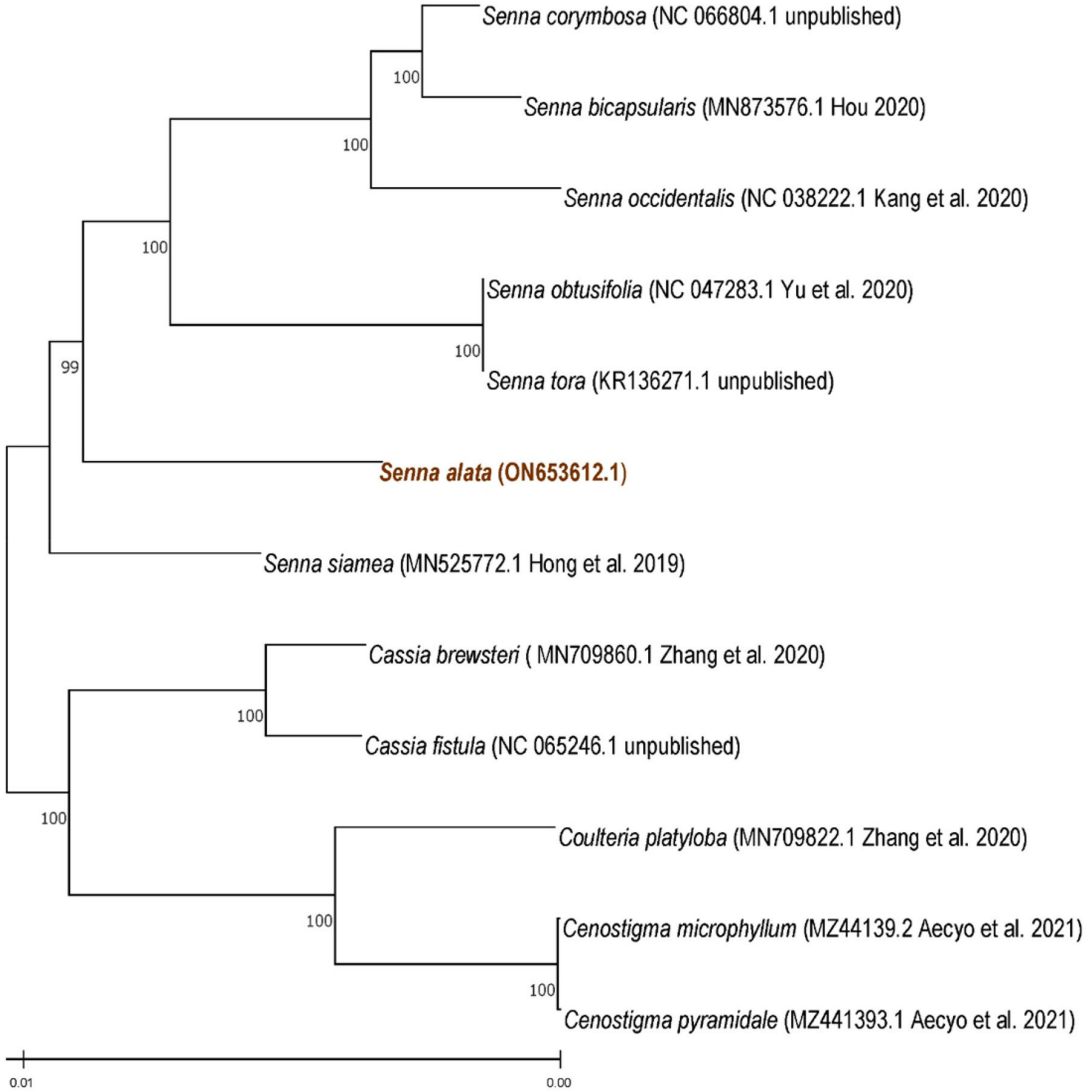
Phylogenetic tree reconstructed using maximum likelihood (ML) method based on complete chloroplast genome sequences of the nine Fabaceae species. Numbers above the lines represent ML bootstrap values (>98%).

The coding sequences from the chloroplast genomes of *S. alata* and twelve other related species (NC 047283.1, KR136271.1, MN873576.1, NC 038222.1, MN525772.1, MN709860.1, MN709822.1, MZ441394.1, MZ441392.1, MZ441393.1, NC_065246.1, NC_066804.1) were downloaded from the NCBI database to construct a phylogenetic tree. These were aligned using MAFFT (Katoh and Standley 2013) and phylogenetic analysis was performed using MEGA-X (Kumar et al. 2018). These methods generated a Maximum Likelihood (ML) tree with the General Time Reversible, Gamma-Invariant (GTR + G + I) as the model (Nei and Kumar, 2000) with 1,000 bootstrap replicates. The phylogenetic analysis showed two major clades, the *Senna* clade and the outgroups including *Cassia* and *Cenostigma* species (Figure 3). The phylogenetic tree also shows a close relationship between *S. alata* and *S. siamea*.

## Conclusion

The complete chloroplast genome of *S. alata* was sequenced and the assembled genome has a sequence length of 159,176 bp which was shorter than the other *Senna* species. Phylogenetic analysis showed a close relationship between *S. alata* and *S. siamea*. The data will contribute to the elucidation of phylogenetic relationships under the genus *Senna* of the Fabaceae family.

## Supplementary Material

Supplemental Material

## Data Availability

The genome sequence data that support the findings of this study are openly available in GenBank of NCBI at https://www.ncbi.nlm.nih.govunder the accession no. ON653612. The associated BioProject, SRA, and Bio-Sample numbers are PRJNA867486, SRR20980268 and SAMN30201905, respectively.
