# Supplementary material for: The complete chloroplast genome of Senna alata (L.) Roxb., an important medicinal plant from the Philippines
Source: Mitochondrial DNA B Resour. 2023 Feb 10;8(2):244–8. doi: 10.1080/23802359.2023.2172973 (PMC9930843; doi:10.1080/23802359.2023.2172973)
Supplement: Supplemental Material [file TMDN_A_2172973_SM7162.docx]

Supplementary Material


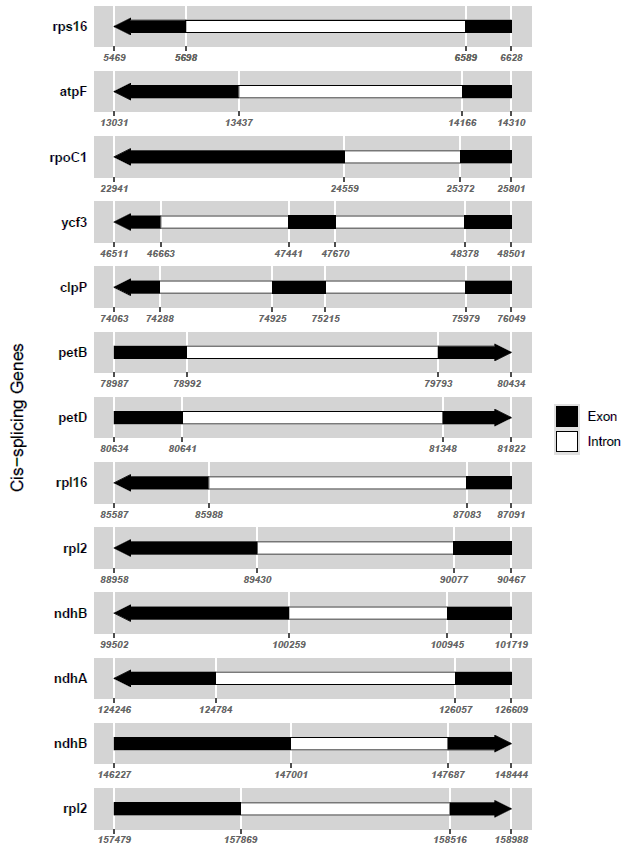


Figure S1. Schematic presentation of cis-splicing genes (CDS) structure in *S. alata* chloroplast genome generated using CPGView. Arrangement of the genes reflect the order represented in the chloroplast genome with names on the left and the structure on the right, with the arrows showing directionality. Exons and introns are represented in black and white, respectively but their lengths are not drawn to scale.


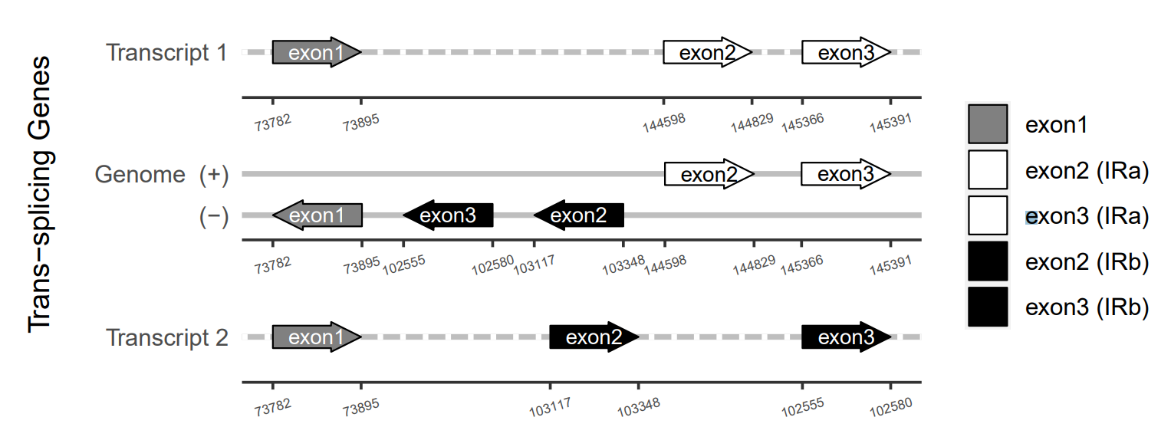


Figure S2. Schematic presentation of trans-splicing genes structure in *S. alata* chloroplast genome generated using GeSeq annotation and CPGView. There are three unique exons found, two of them are duplicated in the IR regions (Note: this trans-spliced region was not annotated in CPGAVAS2)


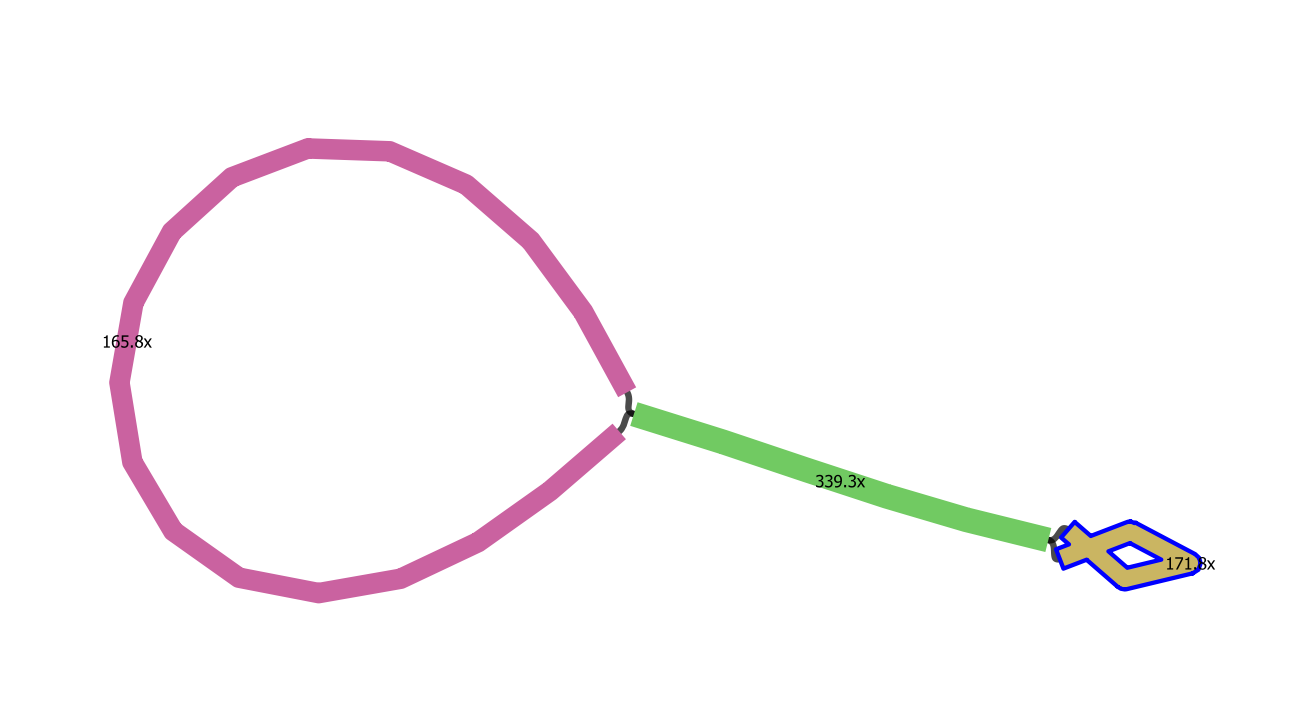


Figure S3. Schematic presentation of the coverage depth of the complete chloroplast genome of *S. alata* using Bandage*.*
